# Intranasal Delivery of Anti-Apoptotic siRNA Complexed with Fas-Signaling Blocking Peptides Attenuates Cellular Apoptosis in Brain Ischemia

**DOI:** 10.3390/pharmaceutics16020290

**Published:** 2024-02-18

**Authors:** Kunho Chung, Irfan Ullah, Yujong Yi, Eunhwa Kang, Gyeongju Yun, Seoyoun Heo, Minkyung Kim, Seong-Eun Chung, Seongjun Park, Jaeyeoung Lim, Minhyung Lee, Taiyoun Rhim, Sang-Kyung Lee

**Affiliations:** 1 Department of Bioengineering and Institute of Nanoscience and Technology, Hanyang University, Seoul 04763, Republic of Korea; chungk@ccf.org (K.C.); yiyujong@gmail.com (Y.Y.); seoyoun1217@gmail.com (S.H.);; 2 Lerner Research Institute, Cleveland Clinic, Cleveland, OH 44106, USA; 3 Department of Internal Medicine, Yale University, New Haven, CT 06520, USA

**Keywords:** ischemic stroke, cell death, apoptosis, Fas signaling, Fas-blocking peptide (FBP), intranasal, siRNA

## Abstract

Ischemic stroke-induced neuronal cell death leads to the permanent impairment of brain function. The Fas-mediating extrinsic apoptosis pathway and the cytochrome c-mediating intrinsic apoptosis pathway are two major molecular mechanisms contributing to neuronal injury in ischemic stroke. In this study, we employed a Fas-blocking peptide (FBP) coupled with a positively charged nona-arginine peptide (9R) to form a complex with negatively charged siRNA targeting Bax (FBP9R/siBax). This complex is specifically designed to deliver siRNA to Fas-expressing ischemic brain cells. This complex enables the targeted inhibition of Fas-mediating extrinsic apoptosis pathways and cytochrome c-mediating intrinsic apoptosis pathways. Specifically, the FBP targets the Fas/Fas ligand signaling, while siBax targets Bax involved in mitochondria disruption in the intrinsic pathway. The FBP9R carrier system enables the delivery of functional siRNA to hypoxic cells expressing the Fas receptor on their surface—a finding validated through qPCR and confocal microscopy analyses. Through intranasal (IN) administration of FBP9R/siCy5 to middle cerebral artery occlusion (MCAO) ischemic rat models, brain imaging revealed the complex specifically localized to the Fas-expressing infarcted region but did not localize in the non-infarcted region of the brain. A single IN administration of FBP9R/siBax demonstrated a significant reduction in neuronal cell death by effectively inhibiting Fas signaling and preventing the release of cytochrome c. The targeted delivery of FBP9R/siBax represents a promising alternative strategy for the treatment of brain ischemia.

## 1. Introduction

Cerebral ischemia, characterized by insufficient blood flow to the brain, remains a global cause of mortality and permanent disabilities. Ischemic stroke, alone, is responsible for over 9.5% of disease-related deaths, affecting more than 15 million individuals [1,2,3]. The primary cause of ischemic stroke is the occlusion of major cerebral arteries, particularly the middle cerebral artery (MCAO), often resulting from thrombus or embolism [4]. The pathological progression of ischemic stroke involves poor glucose storage and anaerobic metabolism, leading to neuronal cell death and permanent disability [5].

Ischemic stroke triggers neuronal cell death through two well-recognized pathways. The Fas-mediated extrinsic apoptosis pathway and the cytochrome c-mediated intrinsic apoptosis pathway both significantly contribute to neuronal injury [6]. The ischemic penumbra in focal ischemia shows an upregulation of Fas-mediated signaling pathways. This observation has prompted investigations into blocking these pathways as a potential strategy to ameliorate neuronal injury caused by ischemic stroke [7,8,9,10,11,12]. Conversely, the intrinsic pathway involves the release of cytochrome c from the mitochondria, with Bax playing a pivotal role in this process. Previous studies have demonstrated that preventing Bax activation provides protection against neuronal apoptosis in ischemic stroke injury [13,14]. Various therapeutic nucleotides, including small interference RNA (siRNA), short hairpin RNA (shRNA), microRNA (miRNA), non-viral plasmid DNA, and viral vectors, have been widely investigated for ischemic stroke treatment [15,16,17,18,19,20,21]. Despite these efforts, the majority of studies have relied on invasive methods for drug delivery to the injured brain tissue.

The delivery of drugs to the brain through the systemic route is impeded by the blood–brain barrier (BBB), characterized by specialized tight junctions among epithelial cells surrounding blood vessels in the brain tissue [22]. Although various approaches, such as glucose transporters or receptor-mediated delivery through transferrin receptors, insulin receptors, leptin receptors, or acetylcholine receptors [23,24,25,26,27,28,29] have been explored, they face limitations in achieving effective drug delivery [30,31,32]. In response to challenges posed by the BBB, intranasal (IN) drug delivery has emerged as a promising and versatile approach, offering a direct and efficient pathway for administering various therapeutic agents [33,34]. This approach bypasses the BBB through the utilization of olfactory and trigeminal nerves and cerebrospinal fluid (CSF) as channels from the nasal cavity to the central nervous systems (CNS) and addresses the limitation associated with traditional routes such as oral or parenteral administration [35]. The nasal mucosa, characterized by its rich vascularization and proximity to the central nervous system, presents an appealing route for delivery, facilitating both local and systemic effects. Intranasal administration offers distinct advantages over conventional routes. The nasal cavity’s expansive surface area and permeable mucosal membrane enable swift absorption and a rapid onset of action. Furthermore, the circumvention of hepatic first-pass metabolism associated with oral administration can enhance bioavailability, allowing for the use of lower doses and reducing systemic exposure. These factors collectively contribute to the potential for enhanced therapeutic efficacy and a more favorable safety profile. Research previously conducted by our group and others has explored intranasal delivery across various therapeutic realms, including anti-cancer drugs, Fas-blocking peptides, insulin, morphine, corticosteroids, and more in the treatment of brain-associated diseases [11,36,37,38,39]. Other studies have investigated the intranasal delivery of therapeutic agents for ischemic stroke treatment, encompassing small chemicals, peptides, proteins, and exosomes [11,40,41,42,43], suggesting intranasal delivery as a valuable tool in modern drug development and therapeutics.

In our previous research, we demonstrated the ability of ligand-conjugated cell-penetrate peptides (CPP) to deliver siRNA through membrane inversion or receptor-mediated endocytosis [29,37,44,45,46,47,48]. Notably, the intranasal delivery of a fusion peptide composed of a neuronal cell-specific ligand and nona-arginine (9R) complexed with siRNA showed promising results, demonstrating specific localization in the brain and notable therapeutic efficacy in a virus-mediated encephalitis model [45]. In this study, we present evidence that the intranasal delivery of a Fas-blocking peptide (FBP) conjugated with a 9R (FBP9R) facilitates siRNA delivery into Fas-expressing apoptotic neurons while sparing normal neurons in brain ischemia. Our FBP9R, combined with anti-apoptotic siRNA targeting Bax involved in the intrinsic apoptosis pathway and administered intranasally, precisely localizes and delivers siBax to Fas-expressing infarcted regions in brain ischemia, resulting in a significant inhibition of ischemic-induced apoptosis. This novel FBP9R/siBax delivery platform effectively blocks apoptosis signaling through both the Fas-mediated extrinsic pathway and the cytochrome c-mediated intrinsic pathway.

## 2. Materials and Methods

### 2.1. Peptide

The peptides used in this study were custom synthesized by Lugen Sci Co. (Bucheon, Gyeonggi-do, Korea). The lyophilized peptide was dissolved in PBS at pH 7.4 and stored frozen at −70 °C for experimental use. The sequences of the synthesized peptides are as follows:

Fas-blocking peptide with 9R (FBP9R): YCDEHFCYGGGGRRRRRRRRRC

Control peptide with 9R (CTR9R): YCNSTVCYGGGGRRRRRRRRRC

### 2.2. siRNA Synthesis

siRNA targeting murine and rat SOD1, Bax, human CD4 and GFP was synthesized by Bioneer (Seoul, Korea). Additionally, FITC-labeled siRNA (siFITC) and Cy5-labeled siRNA (siCy5) targeting the firefly luciferase mRNA were obtained from Bioneer (Seoul, Korea). The sequences of siRNA are as below:

Murine and rat SOD1-targeting siRNA (siSOD1): 5′-GGUGGAAAUGAAGAAAUTAdTdT-3′ [29].

Murine and rat Bax-targeting siRNA (siBax): 5′-CUCCGGCGAAUUGGAGAUGAAdTdT-3′ [49].

GFP-targeting siRNA (siGFP): 5′-GGCUACGUCCAGGAGCGCAdTdT-3′ [44].

Human CD4-targeting siRNA (siCD4): 5′-GAUCAAGAGACUCCUCAGUdTdT-3′ [50].

FITC- or Cy5-labeled siRNA targeting firefly luciferase mRNA (siFITC or siCy5): 5′-CUUACGCUGAGUACUUCGAdTdT-3′ [48].

### 2.3. Gel Retardation Assay

About 100 pmole of siRNA was preincubated for 15 min at room temperature with FBP9RC at peptide/siRNA molar ratios ranging from 0 to 40:1. The samples were mixed with 6× gel loading dye (GenDEPOT, Katy, TX, USA), loaded onto 1% agarose gel, and run at 100 V for 30 min. The intensities of siRNA bands were estimated using Image J software (Version 1.54d), and the complexation percentage and relative siRNA intensity of naked siRNA bands were set to 100%.

### 2.4. Particle Size and Surface Charge Measurement

The zeta average size and surface charge of the peptide/siRNA nanocomplexes were evaluated using a Zetasizer Nano ZS (Malvern Instruments, Worcestershire, UK). The peptides were complexed with 100 pmole of siRNA at various molar ratios in deionized water filtered through a 0.2 μm membrane for 15 min. Subsequently, the peptide/siRNA nanocomplexes were adjusted to a volume of 1 mL with deionized water and filtered through a 0.2 μm membrane for measurement. All data, including Z-average size (nm) and zeta potential (mV) at 25 °C with a measurement angle of 12.8°, were obtained from five replicates. For data analysis, the viscosity (0.8872 mPa·s) and refractive index (1.33) of water were considered to determine Z-average size.

### 2.5. Atomic Force Microscopy

The morphology of peptide/siRNA complexes was investigated using Atomic Force Microscopy (Nano-R2TM AFM, PACIFIC Nanotechnology, Santa Clara, CA, USA). The peptides were complexed with 10 pmole of siRNA at a 20:1 molar ratio in deionized water and incubated for 15 min at room temperature. Approximately, 5 μL of peptide/siRNA complexes was deposited onto the surface of a fresh silicon wafer and rapidly frozen with liquid nitrogen. Subsequently, peptide/siRNA complexes were freeze-dried and prepared for imaging. The obtained images were processed using XEI software (Version 4.3.4).

### 2.6. Cell Culture and In Vitro Hypoxia Induction

The murine neuroblastoma cell line (Neuro-2a; CCL-131, ATCC, Manassas, VA, USA) and human leukemia cell line (Jurkat clone E6-1; KCLB 40152, Korean Cell Line Bank, Seoul, Korea) were obtained from ATCC (Rockville, MD, USA). Neuro-2a cells were cultured in Dulbecco’s Modified Eagle Media (DMEM) supplemented with 10% fetal bovine serum, 1% penicillin (100 IU/mL) and streptomycin (100 μg/mL). To mimic an in vitro ischemia/reperfusion environment, Neuro-2a cells were cultured under hypoxic conditions with 5% CO_2_ and 1% O_2_ using oxygen–glucose deprivation media (Life Technologies, Carlsbed, CA, USA) for 36 h.

### 2.7. Cytotoxicity

To confirm the cytotoxicity of peptide/siRNA complexes on Jurkat and Neuro-2a cells, cell viability was determined using the CCK-8 assay (Dojindo Laboratories, Kumamoto, Japan) following the manufacturer’s instructions. The analysis was conducted 24 h post-transfection with 100 pmole siRNA complexed with the peptide at the indicated peptide/siRNA molar excess.

### 2.8. Evaluation of siRNA Uptake Efficacy In Vitro

To confirm the efficacy of siRNA uptake by peptide/siRNA nanocomplexes, Jurkat and Neuro-2a cells were transfected with 200 pmole of FITC-labeled siRNA (siFITC) complexed with FBP9R at the indicated molar ratio, and lipofectamine 2000 (LMN; Invitrogen, Carlsbad, CA, USA) was used as a positive control. Cells were harvested and washed with PBS 24 h post-transfection. For the analysis of siRNA uptake efficacy, cells fixed with 3% formaldehyde were acquired using FACS Calibur (BD bioscience, Franklin Lakes, NJ, USA). Data analysis was performed using Flowjo software (Version 10.9.0). To confirm the intracellular localization of FITC-labeled siRNA transfected into hypoxic and normoxic Neuro-2a cells, cells were cultured in a chamber-slide system (Life Technologies, Carlsbad, CA, USA) and transfected with 10 pmole of FITC-labeled siRNA complexed with FBP9R at a 20:1 peptide/siRNA molar excess. The cells were then incubated with oxygen–glucose deprivation media or DMEM media containing 50 mM of Lysotracker-DND-99 (Life Technologies, Carlsbad, CA, USA). After washing with PBS and fixation in a 3% formaldehyde solution supplemented with 2% FBS in PBS for 30 min at room temperature, chamber slides were mounted with mounting solution (Abcam, Cambridge, UK) following counterstaining with Hoechst 33342 (Life Technologies, Carlsbad, CA, USA) for 10 min at room temperature. Fluorescence on the chamber slide was captured using a Leica TCS-SP5 confocal microscope (Leica, Wetzlar, Germany) with a 40× objective. Image processing was conducted using Leica X software (Version 3.7.4).

### 2.9. Evaluation of Gene-Silencing Efficacy In Vitro

To confirm the efficacy of gene silencing by the peptide/siRNA nanocomplexes, Jurkat and Neuro-2a cells were transfected with 200 pmole of human CD4 and murine SOD1-targeting siRNA complexed with FBP9R at the indicated molar ratio, and lipofectamine 2000 (LMN; Invitrogen, Carlsbad, CA, USA) was used as a positive control. Then, 24 h post-transfection, cells were harvested and washed with PBS twice. Total mRNA was extracted from the harvested cells using an RNAiso kit (Takara, Kyoto, Japan), reverse-transcribed with the iScript cDNA synthesis kit (Bio-Rad, Hercules, CA, USA), and quantified with SensiFAST SYBR Lo-Rox mix (Bioline, London, UK) on the ABI 7500 Fast Real-time PCR system (Applied Biosystem, Waltham, MA, USA). Human CD4 and murine SOD1 mRNA levels were quantified using the depicted primer sets and normalized to human and murine GAPDH.

### 2.10. Evaluation of Apoptotic Cells

To validate the functional impact of peptide/siRNA complexes in hypoxia Neuro-2a cells, we transfected hypoxic Neuro-2a cells with 300 pmole of Bax-targeting siRNA (siBax) complexed with FBP9R in a 20:1 molar ratio. The cells were then incubated under hypoxic conditions. After 36 h, apoptotic cells were assessed using the Annexin V PE Apoptosis Detection kit (BD bioscience, Franklin Lakes, NJ, USA) following the manufacturer’s instruction. Subsequently, the stained cells were acquired using the FACS Calibur system and analyzed with Flowjo software (Version 10.9.0).

### 2.11. Animal Studies

All experiments were conducted in accordance with guidelines and approved protocols by the Institutional Animal Care and Use Committee (IACUC) at Hanyang University. Sprague–Dawley rats weighing 280–320 g (Orient Bio, Seoul, Korea) were used for the study (*n* = 6 per group). Acute cerebral ischemia was induced by a 1 h occlusion of the right middle cerebral artery (MCAO), following established procedures [51]. Briefly, the ischemic stroke injury was initiated by ligating the external carotid artery (ECA) with a nylon suture, followed by a complete occlusion of the common carotid artery (CCA) using another nylon suture. Reperfusion was initiated after 1 h of occlusion by withdrawing the suture, as previously described [11]. Intranasal administration of peptide/siRNA complexes was carried out using a pressurized olfactory device (Impel Neuropharma, Seattle, WA, USA), as detailed in a previous study [11].

### 2.12. Localization of Intranasally Delivered FBP9R/siRNA

To investigate the localization of intranasally delivered peptide/siRNA nanocomplexes, 2 nmole of Cy5-labeled siRNA, complexed with a CTR9R or FBP9R peptide, was intranasally administrated as a single dose 12 h post-MCAO surgery to each cohort (*n* = 6 per group). Brain tissues were harvested at designated time points for analysis. Fluorescence analysis was conducted on cryosectioned brain slides, which were counterstained with Hoechst 33342. Single-cell suspensions were prepared and examined using a Leica TCS-SP5 confocal microscope and FACS Calibur at specified time points. Images were processed using Leica X software (Version 3.5.5.19976), and FACS data were analyzed using Flowjo software (Version 10.9.0).

### 2.13. Evaluation of Gene-Silencing Efficacy In Vivo

To confirm the silencing efficacy of FBP9R/siRNA complexes, 2 nmole of siRNA targeting SOD1, Bax and GFP, complexed with FBP9R, was intranasally administrated as a single dose 12 h post-MCAO surgery to each cohort (*n* = 6 per group). Total mRNA was extracted from brain tissue using an RNAiso kit (Takara, Kyoto, Japan), reverse transcribed with the iScript cDNA synthesis kit (Bio-Rad, Hercules, CA, USA), and quantified with SensiFAST SYBR Lo-Rox mix (Bioline, London, UK) on the ABI 7500 Fast Real-Time PCR system (Applied Biosystem, Waltham, MA, USA).

### 2.14. Evaluation of Pathology in Brain Tissues

Brain tissue pathology was analyzed by staining 2 mm brain slices in 2% 2,3,5-tryphenyltetrazolium chloride (TTC, Sigma Aldrich, St. Louis, MO, USA), and the infarcted volume was calculated after fixation using Image J software (Version 1.54d), as described previously [11]. Rats subjected to MCAO were intranasally treated with 2 nmole of siBax complexed with FBP9R in a 20:1 molar ratio for functional studies (*n* = 4 per group). A histological analysis of infarcted brain tissues was performed on paraffin-embedded brain sections using H&E staining.

### 2.15. Immunohistochemistry

Immunohistochemistry for cleaved caspase-3 was conducted using an anti-rat cleaved caspase-3 antibody (Abcam, Cambridge, UK) and a secondary polyclonal antibody to rabbit IgG coupled with FITC (Abcam, Cambridge, UK). Fluorescence images were captured with a Leica TCS-SP5 confocal microscope (Leica, Wetzlar, Germany) and processed with Leica X software (Version 3.7.4). Apoptosis in infarcted brain tissue was analyzed using an In Situ Cell Death Detection Kit (Roche, Basel, Switzerland) according to the manufacturer’s instructions. Fluorescence from TUNEL stained signals was captured with a Leica TCS-SP5 confocal microscope and processed with Leica X software (Version 3.7.4). Neurological deficits were evaluated as previously described [11].

### 2.16. Western Blot

To investigate differences in the intracellular localization of cytochrome c by silencing Bax, animals with ischemic stroke were intranasally inoculated in a single dose with 2 nmole of Bax-targeting siRNA (siBax) complexed with FBP9R in a 20:1 molar ratio at 12 h post-infarction. At 48 h post-infarction, the infarcted hemisphere was extracted, and cytosolic and mitochondrial proteins were isolated using the Cytochrome C Release Kit (Abcam, London, UK) according to the manufacturer’s instructions. Cytosolic and mitochondrial lysates from the infracted hemisphere were transferred onto a nitrocellulose transfer membrane from a 15% polyacrylamide gel. The blots were probed with an anti-cytochrome c antibody provided in the Cytochrome C Release Kit and a secondary polyclonal antibody to rabbit IgG coupled to HRP (Abcam, London, UK). The blots were developed using the ECL substrate (Promega, Medison, WI, USA).

### 2.17. Statistical Analysis

Statistical comparisons were conducted using the following methods: for comparisons between two groups at each time point, the Mann–Whitney U Test was employed. To compare more than two groups at each time point, the Kruskal–Wallis Test was utilized. Two-way ANOVA was employed for comparisons among two groups across all time points. Statistical analyses were performed using Graphpad Prism 7 software. A significance level of *p* < 0.05 was considered statistically significant.

## 3. Results

### 3.1. Physical Characterization of FBP9R/siRNA Nanocomplexes

**Figure 1:**
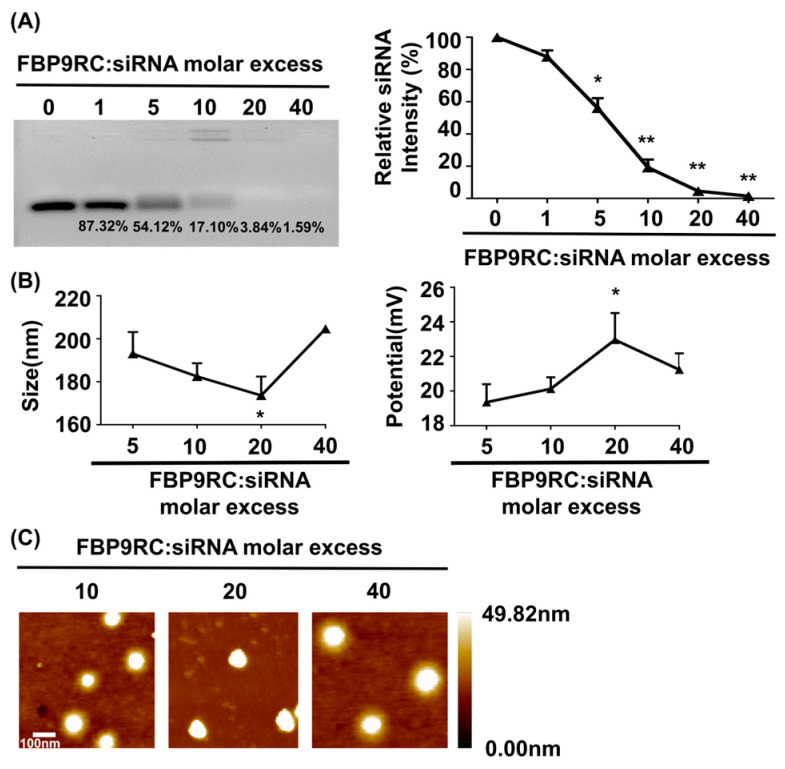
FBP9R peptide enables the formation of nanocomplexes with siRNA. (**A**) Gel retardation assay of the FBP9R peptide and siRNA across various molar ratios. The FBP9R peptide was complexed with 100 pmole of siRNA at the indicated molar excess, and the complexation percentage and relative siRNA intensity were calculated as compared to the naked siRNA group. (**B**) Dynamic light scattering (DLS) analysis of average particle diametric size (right panel) and average surface charge distribution (left panel) of FBP9R/siRNA nanocomplexes. FBP9R peptides were complexed with 100 pmole of siRNA at the indicated molar excess. (**C**) Atomic force microscope (AFM) images illustrating FBP9R/siRNA nanocomplexes at different molar ratios of FBP9R peptide and siRNA. The scale bar indicates 100 μm. Mann–Whitney U Test was used for statistical analysis versus the 5:1 group. * *p* < 0.05, ** *p* < 0.01.

The physical characterizations of FBP9R/siRNA nanocomplexes are presented in Figure 1, which demonstrates a thorough investigation into the properties of Fas-targeting delivery systems. FBP9R successfully condenses with siRNA at a 20:1 peptide/siRNA molar excess (Figure 1A). The charge and potential assessment revealed that FBP9R forms nanosized complexes with siRNA ranging from 170 nm to 210 nm in diameter, maintaining an average positive charge (Figure 1B). The average surface zeta potential of FBP9R/siRNA at a 20:1 molar ratio surpassed that of other tested peptide/siRNA molar ratios. However, an increase in peptide amount to 40:1 resulted in a decrease in surface zeta potential, indicating a potential for loose complexation, as described in a previous study (Figure 1B) [52]. Furthermore, the morphological analysis using atomic force microscopy (AFM) imaging confirmed the formation of a homogenous nanosized complex of FBP9R/siRNA at all tested molar ratios (Figure 1C). These findings support the conclusion that FBP9R peptide facilitates the formation of nanosized complexes with siRNA and can be an siRNA carrier, specifically to Fas-expressing cells as FBP has the potential to strongly bind to the Fas receptor.

### 3.2. FBP9R/siRNA Delivers siRNA and Induces Target Gene Silencing in Fas-Expressing Cells

**Figure 2:**
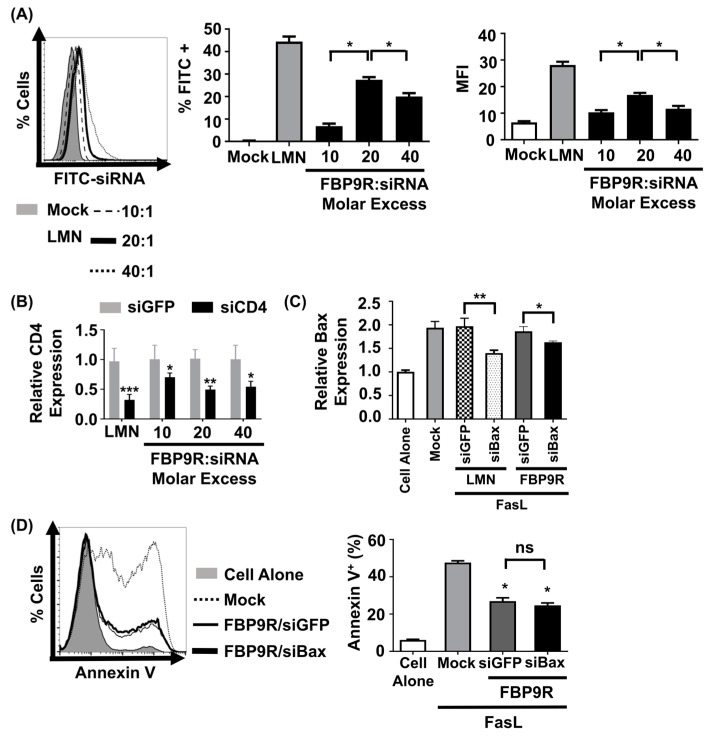
FBP9R enables intracellular delivery of siRNA into Fas-expressing cells. (**A**) Flow cytometry analysis for efficacy of siRNA delivery by FBP9R/siRNA nanocomplexes. FBP9R peptides were complexed with 200 pmole of FITC-labeled siRNA at the indicated peptide/siRNA molar excess. Representative histogram (left panel) and cumulative data for percent of FITC-positive cells (middle panel) and mean fluorescence intensity (right panel). * *p* < 0.05, versus mock group. (**B**) Gene-silencing efficacy of FBP9R/siRNA nanocomplexes in Jurkat cells at indicated peptide/siRNA molar ratios. FBP9R peptides were complexed with 200 pmole of siRNA at the indicated peptide/siRNA molar excess. * *p* < 0.05, ** *p* < 0.01, *** *p* < 0.001 versus siGFP group. Data obtained from three independent experiments, presented as mean ± SD. (**C**) Bax silencing efficacy of FBP9R/siBax nanocomplexes in FasL-treated Jurkat cells. FBP9R peptides were complexed with 200 pmole of siBax in a 20:1 molar ratio and transfected into FasL-treated Jurkat cells. Data collected from three independent experiments, represented as mean ± SD. Statistical analysis by Mann–Whitney U test. * *p* < 0.05, ** *p* < 0.01. (**D**) Anti-apoptotic effect of FBP9RC/siBax on FasL-treated Jurkat cells. Representative histogram indicates flow cytometry analysis of Annexin V stained cells (left panel, gray-cell alone, dotted line-Mock, solid line-FBP9RC/siGFP, thick solid line-FBP9RC/siBax) and cumulative data (light panel) for percent of Annexin V-positive cells were obtained from three independent experiments, including three individual batches, shown as mean ± SD. * *p* < 0.05 versus mock, ns—not significant. Mann–Whitney U Test was used for statistical analysis. FBP—Fas blocking peptide, siGFP—siRNA targeting GFP, siBax—siRNA targeting Bax gene, LMN—Lipofectamin 2000.

To evaluate the functionality of the FBP9R/siRNA nanocomplexes, siRNA uptake and gene-silencing effects were investigated in Jurkat cells, known for their continuous expression of Fas on their surface [11]. Prior to functional assays, cytotoxicity was assessed at varying molar ratios, ranging from 10:1 to 40:1. Results indicated minimal (<15%) cytotoxicity for FBP9R/siRNA up to a 20:1 molar ratio in Jurkat cells. However, at a 40:1 ratio, approximately 46% cytotoxicity was observed (Supplementary Figure S1). In consistence with previous reports, transfection with lipofectamine showed severe toxicity in Jurkat cells. Next, to assess its delivery efficiency, Jurkat cells were transfected with various FBP9R/siRNA molar ratios. Flow cytometry data illustrated the successful delivery of FBP9R/siFITC into Jurkat cells (Figure 2A). The analysis revealed a significant increase in both transfected cell percentages (~28%) and mean fluorescence intensity (MFI) at the 20:1 peptide/siRNA molar ratio compared to other tested ratios (Figure 2A). These data indicate the superior delivery of FBP9R at the 20:1 molar ratio. Consistent with the siRNA delivery results, FBP9R/siCD4 exhibited a significant reduction in the target CD4 gene by an average of ~50% in Jurkat cells at the 20:1 molar ratio (Figure 2B). This finding underscores the gene-silencing capability of FBP9R/siRNA complexes. After confirming that the 20:1 molar ratio of FBP9R/siRNA provides optimal delivery, we used this ratio in all subsequent experiments. We then evaluated the functionality of FBP9R to deliver siBax to cells treated with FasL to induce Fas-mediated apoptosis conditions. FBP9R facilitated the significant downregulation of Bax in FasL-treated Jurkat cells compared to FasL-treated cells without treatment (Figure 2C). However, despite the effective downregulation of Bax, it did not lead to a reduction in Fas-mediated apoptosis when compared to siGFP (Figure 2D). Our data demonstrate that FBP9R can deliver siRNA to Fas expressing cells.

### 3.3. FBP9R/siRNA Effectively Delivers siRNA and Induces GENE Silencing in Hypoxia-Induced Neuro-2a Cells

**Figure 3:**
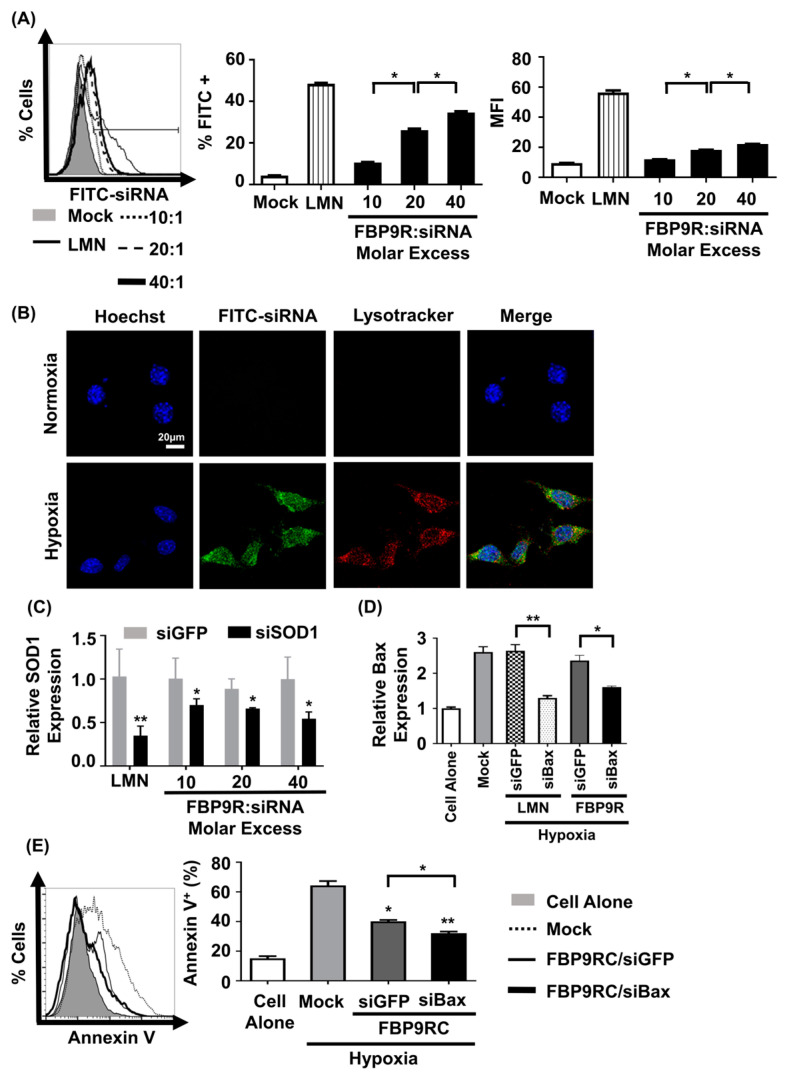
FBP9R enables intracellular translocation of siRNA into hypoxia-induced Neuro-2a cells. (**A**) Flow cytometry analysis for efficacy of siRNA delivery by FBP9R/siRNA nanocomplexes in hypoxia Neuro-2a cells. FBP9R peptides were complexed with 200 pmole of FITC-labeled siRNA in indicated peptide/siRNA molar excess. Representative histogram (left panel) and cumulative data for percent of FITC-positive cells (middle panel), mean fluorescence intensity (right panel). * *p* < 0.05, versus mock group. (**B**) Intracellular translocation of siRNA by FBP9R/siRNA nanocomplexes in normoxia (upper panel) and hypoxia (lower panel) Neuro-2a cells. Fluorescence distribution of FITC-labeled siRNA (green), endosomal vesicle stained with lysotracker (red) and nuclei counterstained with Hoechst 33342 (blue) are shown. The scale bar indicates 20 μm. (**C**,**D**) Gene-silencing efficacy of FBP9R/siRNA nanocomplexes in hypoxia Neuro-2a cells at various peptide/siRNA molar ratios. FBP9R peptides were complexed with 300 pmole of siRNA at the indicated peptide/siRNA molar excess. * *p* < 0.05, ** *p* < 0.01 versus siGFP group. (**E**) Knockdown of Bax by FBP9R/siBax nanocomplexes in hypoxia Neuro-2a cells. * *p* < 0.05, ** *p* < 0.01 versus mock. All data were obtained from three independent experiments and shown as mean ± SD. Mann–Whitney U test was used for statistical analysis. FBP—Fas blocking peptide, siGFP—siRNA targeting GFP, siFITC—siRNA targeting FITC, siSOD1—siRNA targeting superoxide dimutase gene 1, siBax—siRNA targeting Bax gene, LMN—Lipofectamin 2000.

We next assessed siRNA uptake and gene-silencing effects by FBP9R/siRNA in hypoxic Neuro-2a cells to mimic the in vitro ischemic condition [11]. The nanocomplexes exhibited no significant cytotoxicity up to a 40:1 molar ratio on Neuro-2a cells (Supplementary Figure S2). Consistent with the Jurkat cell data, FBP9R/siFITC efficiently delivered siFITC, evident from the percentage of FITC-positive cells and induced gene silencing in cells transiently expressing Fas under the hypoxic condition (Supplementary Figure S3). Approximately 37% of cells were positive for siFITC by FBP9R/siRNA in Fas-positive hypoxic cells (Figure 3A), with no significant uptake observed in normal Neuro-2a cells (Supplementary Figure S4). Confocal microscopy data revealed the significant colocalization of FBP9R/siFITC nanocomplexes with endosomal vesicle stained with lysotracker 24 h post-transfection in hypoxia Neuro-2a cells, compared to normoxic Neuro-2a cells (Figure 3B). This suggests efficient intracellular trafficking and the endosomal escape of the nanocomplexes to Fas-expressing hypoxic cells. Consistent with the siRNA delivery results, FBP9R/siRNA nanocomplexes downregulated gene expression by approximately 44% of SOD1 and 40% of Bax in hypoxic Neuro-2a cells at a 20:1 molar ratio (Figure 3C). Additionally, FBP9R/siBax treatment resulted in a two-fold decrease in Annexin V-positive cells compared to hypoxic Neuro-2a cells (Figure 3E). The observed reduction in apoptosis in FBP9R/siGFP is possibly attributed to the functional effects of the FBP peptide itself, as previously reported [11,37]. Interestingly, we observed additive protective effects with FBP/siBax, suggesting that reducing Bax levels could reduce apoptosis. The data suggest that FBP9R/siBax specifically targets Fas-expressing cells and improves hypoxia-induced apoptosis in Neuro-2a cells by lowering Fas- as well as Bax-mediated apoptosis pathways (Figure 3E). Therefore, this strategy is considered for the delivery of siBax to a cerebral ischemia model in vivo.

### 3.4. Intranasally Administrated FBP9R Successfully Delivers siRNA and Enables Gene Silencing in the Infarcted Hemisphere of a Rat Model of MCAO

**Figure 4:**
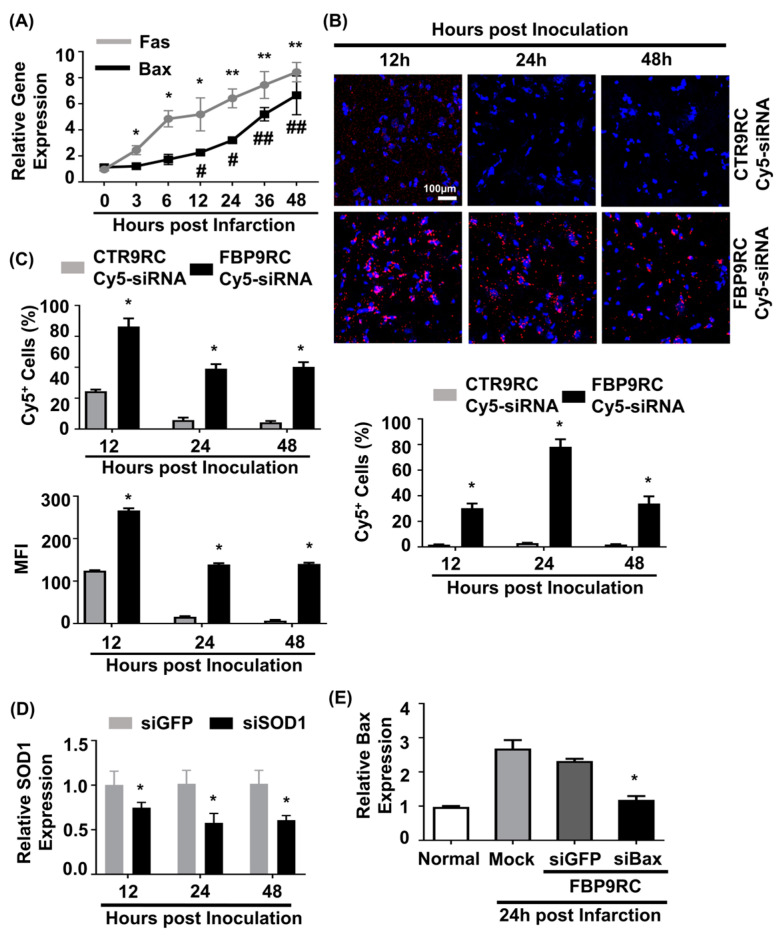
Intranasally delivered FBP9R/siRNA nanocomplexes specifically localize to Fas-expressing brain regions in the rat model of brain ischemia. (**A**) Gene expression profile of Fas and Bax depending on hours post-MCAO surgery. * *p* < 0.05 and ** *p* < 0.01 versus 0 h post-infarction. ^#^ *p* < 0.05 and ^##^ *p* < 0.01 versus 0 h post-infarction. (**B**) Immunohistochemistry of intranasally delivered Cy5-labeled siRNA in the infarcted hemisphere at indicated hours post-injection. Representative images (upper panel) and cumulative data from six independent animals per group (lower panel) indicate the percent of Cy5-positive cells. The scale bar indicates 100 μm. * *p* < 0.05 versus CTR9R group. (**C**) Flow cytometry analysis of Cy5-positive cells in the infarcted hemisphere. Cumulative data for percent of Cy5-positive cells (upper panel) or mean fluorescence intensity (lower panel) obtained from six independent animals per group. * *p* < 0.05 versus sham group. (**D**) Gene-silencing efficiency of intranasally delivered FBP9R/siRNA nanocomplexes in infarcted hemisphere from six independent animals. * *p* < 0.05 versus FBP9R/siGFP. (**E**) Silencing efficiency of FBP9R/siBax nanocomplexes on 24 h post-intranasal administration in the infarcted hemisphere. * *p* < 0.05 versus mock group. Mann–Whitney U Test was used for statistical analysis.

In our previous work, we demonstrated that elevated Fas expression in the ischemic region of rats undergoing MCAO and FBP was shown to specifically bind to Fas following IN administration [11]. In this study, we aimed to simultaneously target both the Fas-mediated extrinsic apoptotic pathway using FBP9R and the Bax-associated intrinsic apoptotic pathway using siBax. Gene expression analysis revealed an upregulation of both Fas and Bax (approximately eight-fold after 48 h of ischemic onset) exclusively in the ischemic region, but not in the control, non-ischemic region (Figure 4A). To investigate the delivery of FBP9R/siRNA complexes in the ischemic region of MCAO, a single IN delivery of FBP9R complexed with Cy5-labeled siRNA (siCy5) 12 h post-ischemic onset was performed. Confocal microscopy data demonstrated a specific deposition of FBP9R/siCy5 complexes into the ischemic region within 12 h post-inoculation, in contrast to the control peptide (CTR9R) (Figure 4B, Supplementary Figure S5A). Flow cytometry analysis of single-cell suspensions from ischemic or non-ischemic regions further confirmed the ischemic-specific entry of FBP9R/siCy5 complexes, with a strong siCy5-positive population observed within the ischemic region (Figure 4C). Approximately, 72%, 42% and 40% of Cy5-positive cells were estimated at 12 h, 24 h and 48 h, respectively, post-delivery with FBP9R. Notably, CTR9R/siCy5 complexes showed a non-specific siCy5-positive population (~22%) at the initial 12 h post-inoculation, but this positivity was negligible at later time points, indicating the drainage of non-internalized siRNA from the brain to the periphery (Supplementary Figure S5B). In contrast, FBP9R/siCy5 complexes specifically localized within the ischemic region and remained there up to 48 h, supporting Fas-specific siRNA delivery, as observed in confocal microscopy data. Subsequently, a single IN inoculation of FBP9R/siSOD1 resulted in a 23%, 46% and 43% knockdown of SOD1 at 12 h, 24 h and 48 h post-inoculation, respectively, in the infarcted hemisphere (Figure 4D). Poor knockdown of SOD1 was observed in the normal hemisphere within the same time frame (Supplementary Figure S5C). This demonstrated that intranasally administered FBP9R/siRNA specifically deposited and induced functional gene silencing in the ischemic brain region of the MCAO model. Furthermore, the therapeutic efficacy was assessed by targeting the Bax gene in the MCAO model. IN administration as a single dose of FBP9R/siBax led to a ~47% knockdown of Bax after 24 h post-inoculation (Figure 4E). These results indicate the potential of FBP9R-mediated siRNA delivery for targeted gene silencing in the ischemic brain, highlighting its potential therapeutic application in the context of cerebral ischemia.

### 3.5. FBP9R/siBax Attenuates the Infarcted Region and Apoptosis in the Infarcted Hemisphere in a Rat Model of Brain Ischemia

**Figure 5:**
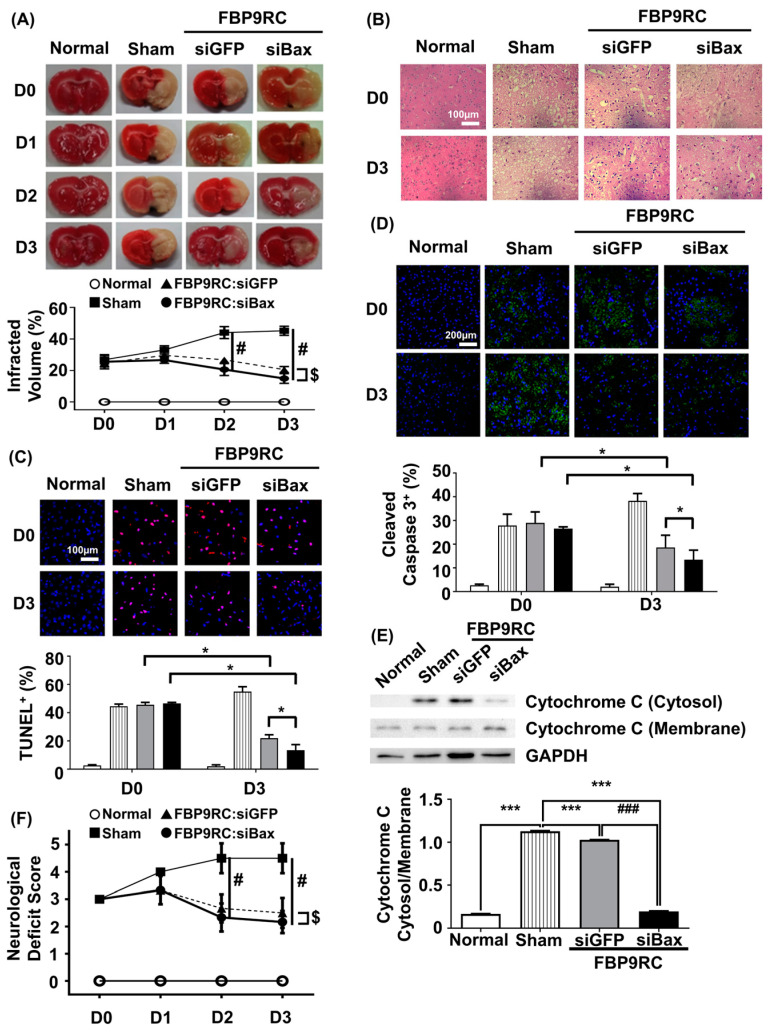
Attenuation of apoptosis in the infarcted hemisphere by intranasally delivered FBP9R/siBax nanocomplexes in a rat model of brain ischemia. (**A**) TTC-stained brain tissue slices after ischemia and reperfusion. Quantitative analysis of representative images is shown below from six independent animals. Two-way ANOVA versus sham group. # *p* < 0.05 versus Sham, ^$^ *p* < 0.05 versus FBP9RC/siGFP. (**B**) Representative images of histology in brain tissue sections from the infarcted hemisphere from six independent animals. The scale bar indicates 100 μm. (**C**) Representative TUNEL-stained brain tissue sections (upper panel) show TUNEL-positive cells (red) and Hoechst-stained nuclei (blue) in the infarcted hemisphere. The scale bar indicates 100 μm. Cumulative analysis of the representative images is shown from six independent animals (lower panel). Mann–Whitney U Test was used for statistical analysis versus the sham group. * *p* < 0.05. (**D**) Immunohistochemistry of cleaved caspase 3 in the infarcted hemisphere on day 0 and day 3 post-injection. Representative images (upper panel) and cumulative quantification analysis (lower panel) obtained from six independent animals. Mann–Whitney U Test was used for statistical analysis versus sham group. * *p* < 0.05. (**E**) Western blot for cytosol presence in the membrane or cytosol in the infracted hemisphere from 3 days post-infarction. Representative western blot (upper panel) normalized into GAPDH. Cumulative analysis for band intensity obtained from six independent animals. Mann–Whitney U Test was used for statistical analysis. *** *p* < 0.001 versus Sham, ### *p* < 0.001 versus FBP9RC/siGFP. (**F**) Neurological deficit score at indicated days after the injection of FBP9R/siBax nanocomplexes from six independent animals. Kruskal–Wallis Test versus sham group. The data represent mean ± SD and # *p* < 0.05 versus Sham, ^$^ *p* < 0.05 versus FBP9RC/siGFP.

Finally, we tested the therapeutic potential of FBP9R/siBax in the context of reversing the ischemic stroke condition in a MCAO rat model. FBP9R/siRNA complexes and 2 nmole of siRNA were intranasally administrated as a single dose at 12 h post-MCAO surgery. The analysis of TTC-stained coronal brain slices after necropsy revealed reduced brain damage in FBP9R/siBax-treated animals compared to sham-operated animals (Figure 5A). In sham-operated animals, an estimated 36%, 47% and 51% brain infarction was observed at day 1, 2 and 3 post-MCAO, respectively, while in FBP9R/siBax-treated groups, this infarction was controlled at 29%, 18% and 13%, respectively (Figure 5A). Both FBP9R/siGFP- and FBP9R/siBax-treated animals showed infarction recovery but targeting Bax in the form of FBP9R/siBax demonstrated better outcomes. Consistent with TTC data, a histopathological examination of treated MCAO brain slices revealed the significant recovery of ischemic-induced tissue damage in both FBP9R/siGFP- and FBP9R/siBax-treated animals (Figure 5B). Subsequently, the therapeutic effect of FBP9R/siBax on overall apoptosis reduction and the inhibition of cleaved caspase activity—factors predominately induced after ischemic onset—was evaluated. Confocal data indicated that apoptotic (TUNEL-stained) and cleaved caspase signals significantly increased within 12 h (shown as D0) and tended to increase until day 3 post-MCAO. However, FBP9R/siBax treatment significantly reduced these effects (Figure 5C,D). At day 3 post-MCAO, an estimated 52% and 74% reduction in TUNEL-positive cells and 31% and 47% reduction in cleaved caspase-3 positivity was observed in FBP9R/siGFP- and FBP9R/siBax-treated animals, respectively (Figure 5C,D). Additionally, FBP9R/siBax treatment led to a significant reduction in the intrinsic apoptosis pathway, as evidenced by the decreased cytosolic cytochrome on day 3 post-MCAO (Figure 5E). Neurological deficit score data also confirmed improvement in and better recovery in the FBP9R/siBax-treated cohort (Figure 5F). In line with histologic and molecular analysis, FBP9RC/siBax treatment improved the neurological deficit score compared to the FBP9R/siGFP group (Figure 5F). Thus, it was concluded that FBP9R-mediated siBax delivery to the ischemic hemisphere exhibits a synergistic effect, inhibiting both extrinsic and intrinsic apoptosis in brain ischemia.

## 4. Discussion

In this study, we introduced an siRNA delivery platform to target Fas-mediated apoptosis (extrinsic apoptosis pathway) and one that targets Bax (intrinsic apoptosis pathway). We observed that Fas-blocking peptide (FBP)-conjugated 9R effectively delivered the siRNA to cultured cells and to the brain after intranasal inoculation, as shown in prior studies where FBP9R complexed with siRNA formed ~200 nm nanocomplexes, serving as a suitable carrier for intranasal delivery to the brain [53,54]. Our findings demonstrate that this system can be used to deliver siRNA to cell types expressing the Fas receptor on their surfaces.

Having established the efficacy of the FBP9R-mediated siRNA delivery system in targeting Fas-mediated apoptosis and Bax, we investigated the therapeutic implications in the context of cerebral ischemia modeled by the MCAO model. Cerebral ischemia is characterized by an intricate cascade of events following an ischemic injury that involve apoptotic pathways [37,55,56]. In our study, we highlight the therapeutic effects of FBP9R, a targeted siRNA delivery system, in the MCAO ischemic stroke model. Leveraging the specificity of FBP9R in binding to Fas-expressing cells, we utilized it as a carrier for delivering siRNA-targeting Bax. Bax, a mediator of the intrinsic apoptotic pathway, plays a critical role in neuronal cell death post-ischemia, exacerbating apoptotic cascades and contributing to secondary brain injury in the ischemic environment. By specifically targeting Bax with siRNA, our approach sought to interrupt these detrimental processes, aiming for a neuroprotective outcome.

The choice of intranasal delivery is particularly significant in the MCAO model, where time is of the essence. This non-invasive route provides a direct conduit to the central nervous system, bypassing the BBB and facilitating rapid access to the ischemic site. Intranasal administration shows promise in optimizing drug bioavailability in the brain, making it an ideal approach for time-sensitive conditions like ischemic stroke [57,58]. Unlike invasive approaches such as intracranial and stereotactic injections, intranasal administration also provides brain-specific drug delivery in a non-invasive manner with reduced side effects [59,60,61]. Numerous studies have previously explored intranasal delivery for ischemic stroke treatment, encompassing various substances, from small chemicals to peptides, proteins, and exosomes [11,40,41,42,43,62]. In particular, the intranasal delivery of siRNA has shown promise in preventing neuronal injuries in ischemic conditions [63]. Previous work emphasizes the significance of the targeting moiety for the sustained localization of intranasally administered drugs in the brain [11,45]. It is noteworthy that mucociliary clearance plays a crucial role in the nasal cavity, significantly influencing the effectiveness of intranasal drug delivery, including drugs or nanocomplexes such as FBP9R/siBax. Successful intranasal drug delivery relies on the drug’s residence time within the nasal cavity. Rapid clearance by the mucociliary system can shorten the drug’s contact with the nasal mucosa, thereby reducing its absorption and bioavailability [64]. Some studies suggest that the nanocomplex may evade mucociliary clearance, potentially prolonging drug release [65,66]. This underscores the importance of better understanding mechanisms that can enhance nasal drug delivery efficacy.

Despite the active exploration of intranasal delivery in human clinical trials for treating brain-associated disorders [11,67,68], translating pre-clinical success in animal models into clinical efficacy faces challenges due to anatomical differences in the nasal cavities of rodents and humans. Specifically, the nasal cavity’s relative surface area in mice and rats is notably higher than that of humans, influencing the efficacy of the intranasal delivery approach. Moreover, the olfactory epithelium, expressed as a percentage of the nasal cavity surface area, is substantially greater in rodents. These anatomical distinctions pose difficulties in accurately determining effective intranasal drug dosing for human application, especially with experimental therapeutics like FBP9R/siRNA. However, recent technical advancements such as mucosal flap reconstruction and pressurized olfactory devices show promise in enhancing drug deposition beyond the nasal valve [69,70]. Continued exploration of such techniques may enhance the precision and clinical success of intranasal delivery for brain-associated disorders.

The pathophysiology of brain damage following ischemic onset involves intricate signaling pathways, encompassing neuronal apoptosis and neuroinflammation [71]. Neuronal cells undergo apoptosis and necrosis due to oxidative stress and neuroinflammation in ischemic conditions [72,73,74]. Investigating molecular factors associated with apoptosis and inflammatory pathways in ischemic stroke has led to the identification of potential therapeutic targets. Studies have explored blocking Fas-mediated signaling to prevent primary ischemic injury and secondary inflammatory injury blocking Fas or FasL [11,37,56,72,75]. Additionally, the downregulation of inflammatory or apoptosis signaling by targeting genes involved in the pathway, such as HIF-1α, Caspase-3, JAK2/STAT3 and HMGB1, has been extensively investigated [15,63,76,77]. Targeting both extrinsic and intrinsic apoptosis pathways has demonstrated improved systolic function in myocardial infarction conditions by applying cowpox virus protein, crmA, at reperfusion [78]. Our results with FBP9R/siBax support the potential of this new therapeutic platform for treating ischemic stroke by concurrently blocking the Fas-mediated extrinsic and Bax-involved intrinsic apoptosis pathways.

Our study represents a pioneering demonstration in two critical aspects: the specific delivery and gene silencing by siRNA in the infarcted region, and the enhanced prevention of apoptosis in ischemic stroke through dual-targeting Fas-mediated extrinsic and Bax-involved intrinsic apoptosis pathways. Intranasal delivery of siRNA using the FBP9R peptide resulted in localized and functionally effective therapeutic siRNA delivery to the Fas-expressing ischemic core of the infarcted brain tissue. The prevention of the Fas-mediated extrinsic apoptosis pathway through blocking Fas signaling, coupled with the downregulation of the Bax-involved intrinsic apoptosis pathway by FBP9R/siBax nanoparticles, led to the augmented prevention of cellular apoptosis in ischemic stroke. These findings underscore the potential of FBP9R/siBax to function as an innovative and effective therapeutic strategy for ischemic stroke, offering a dual-targeted approach to alleviate the complex cellular responses associated with ischemic brain injury. Future studies can further explore the translational potential of this approach and investigate its application in clinical settings.

## 5. Conclusions

Our study establishes FBP9R/siRNA nanocomplexes as robust and efficient siRNA delivery systems, demonstrating promising results in the treatment of brain ischemia. The specificity of FBP9R/siRNA for Fas-expressing cells, coupled with successful gene-silencing and therapeutic efficacy in the rat model of MCAO, highlights the potential of this approach for targeted interventions in brain ischemia. Our data demonstrated synergistic neuroprotection by targeting both the Fas-mediated extrinsic and Bax-involved intrinsic apoptotic pathways. These results provide novel therapeutic platforms for treating cellular apoptosis that occurs in ischemic stroke. Further studies are warranted to explore additional applications and to optimize the delivery system for clinical translation.

## Data Availability

All data are present within the manuscript and Supplementary Materials.

## Supplementary Materials

The following supporting information can be downloaded at: https://www.mdpi.com/article/10.3390/pharmaceutics16020290/s1, Figure S1: Cytotoxicity measured by the CCK-8 viability assay of Jurkat cells; Figure S2: Cytotoxicity measured by the CCK-8 viability assay of Neuro-2a cells; Figure S3: Fas induction in Neuro-2a cells under hypoxic conditions; Figure S4: Ineffectiveness of FBP9R in delivering siRNA into normoxia Neuro-2a cells; Figure S5: Lack of localization of intranasally delivered FBP9R/siRNA nanocomplexes to normoxia brain regions in the rat model of brain ischemia.

## Abbreviations

FBP	Fas-blocking peptide
9R	Nona-arginine peptide
IN	Intranasal
MCAO	Middle cerebral artery occlusion
siRNA	Small interference RNA
shRNA	Short hairpin RNA
miRNA	MicroRNA
BBB	Blood-brain barrier
CSF	Cerebrospinal fluid
CNS	Central nervous systems
CPP	Cell-penetrate peptides
FBP9R	Fas-blocking peptide with 9R
CTP9R	Control peptide with 9R
siFITC	FITC-labeled siRNA
siCy5	Cy5-labeled siRNA
siSOD1	SOD1-targeting siRNA
siBax	Bax-targeting siRNA
siGFP	GFP-targeting siRNA
siCD4	CD4-targeting siRNA
ECA	External carotid artery
CCA	Common carotid artery
TTC	2,3,5-tryphenyltetrazolium chloride
AFM	Atomic force microscopy
DLS	Dynamic light scattering
MFI	Mean fluorescence intensity
