# Supplementary material for: Intranasal Delivery of Anti-Apoptotic siRNA Complexed with Fas-Signaling Blocking Peptides Attenuates Cellular Apoptosis in Brain Ischemia
Source: Pharmaceutics. 2024 Feb 18;16(2):290. doi: 10.3390/pharmaceutics16020290 (PMC10892455; doi:10.3390/pharmaceutics16020290)
Supplement: Supplementary file 1 [file pharmaceutics-16-00290-s001.zip › pharmaceutics-2871294-supplementary.pdf]

Supplementary Materials

# Intranasal delivery of anti-apoptotic siRNA complexed with Fas-signaling blocking peptides attenuates cellular apoptosis in brain ischemia

**Kunho Chung[1,2,†], Irfan Ullah[1,3,†], Yujong Yi[1], Eunhwa Kang[1], Gyeongju Yun[1], Seoyoun Heo[1], Minkyung Kim[1], Seong-Eun Chung[1], Seongjun Park[1], Jaeyeoung Lim[1], Minhyung Lee[1], Taiyoun Rhim[1,*] and Sang-Kyung Lee[1,*]**

**Total Supplementary Figures (S1~S5)**

Figure S1. Cytotoxicity measured by the CCK-8 viability assay of Jurkat cells

Figure S2. Cytotoxicity measured by the CCK-8 viability assay of Neuro-2a cells

Figure S3. Fas induction in Neuro-2a cells under hypoxic conditions

Figure S4. Ineffectiveness of FBP9R in delivering siRNA into normoxia Neuro-2a cells

Figure S5. Peptide toxicity test by CCK-8 assay in ARPE-19 cells

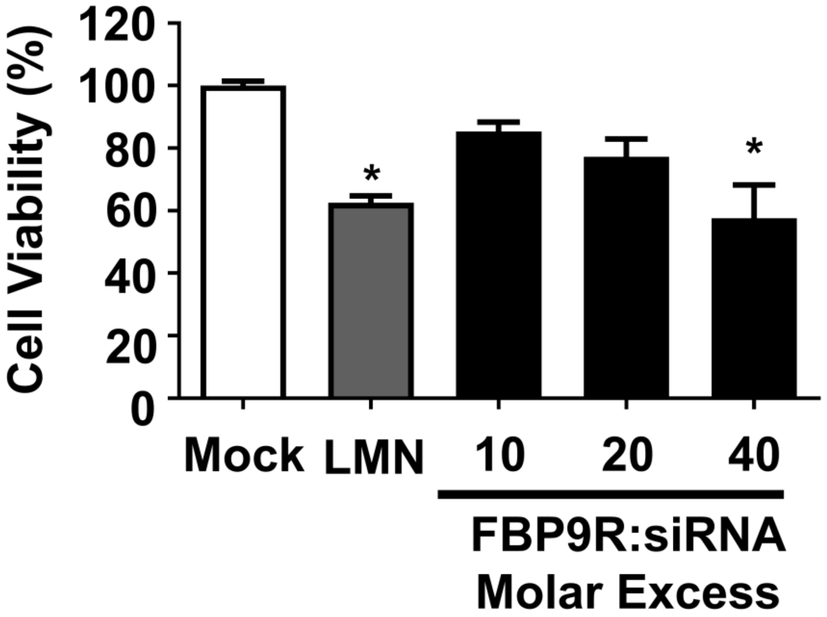


**Figure S1. Cytotoxicity measured by the CCK-8 viability assay of Jurkat cells.** Cytotoxicity assessed 24 hr post-transfection of Jurkat cells with FBP9RC/siRNA nanocomplexes. FBP9RC peptides were complexed with 100 pmole of siRNA at the indicated peptide:siRNA molar excess. Relative percent cell viability was normalized with the mock group. *$p<0.05$ verse 10 group.

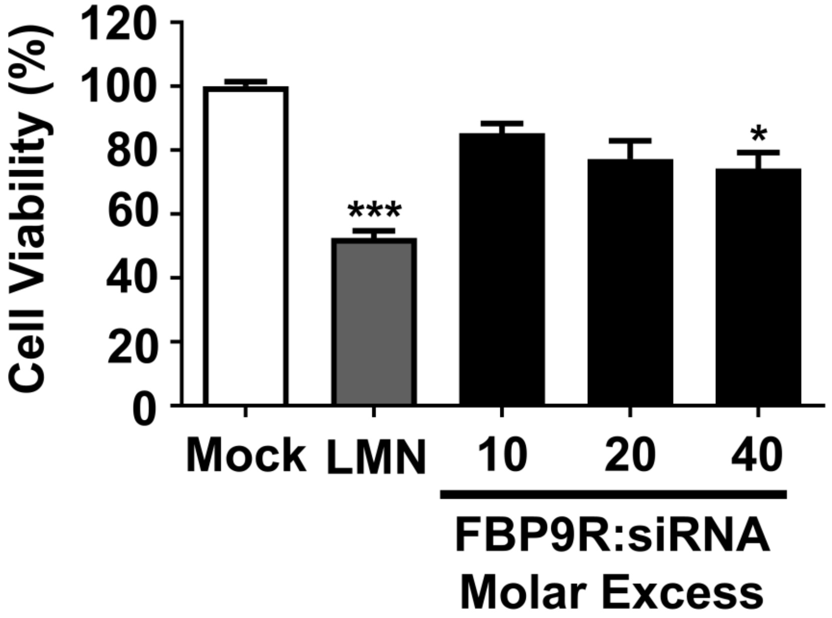


**Figure S2. Cytotoxicity measured by the CCK-8 viability assay of Neuro-2a cells**. Cytotoxicity assessed 24 hr post transfection of Neuro-2a cells with FBP9RC/siRNA nanocomplexes. FBP9RC peptides were complexed with 100 pmole of siRNA at the indicated peptide:siRNA molar excess. Relative percent cell viability was normalized with mock group. *$p<0.05$, ***$p<0.001$ verse 10 group.

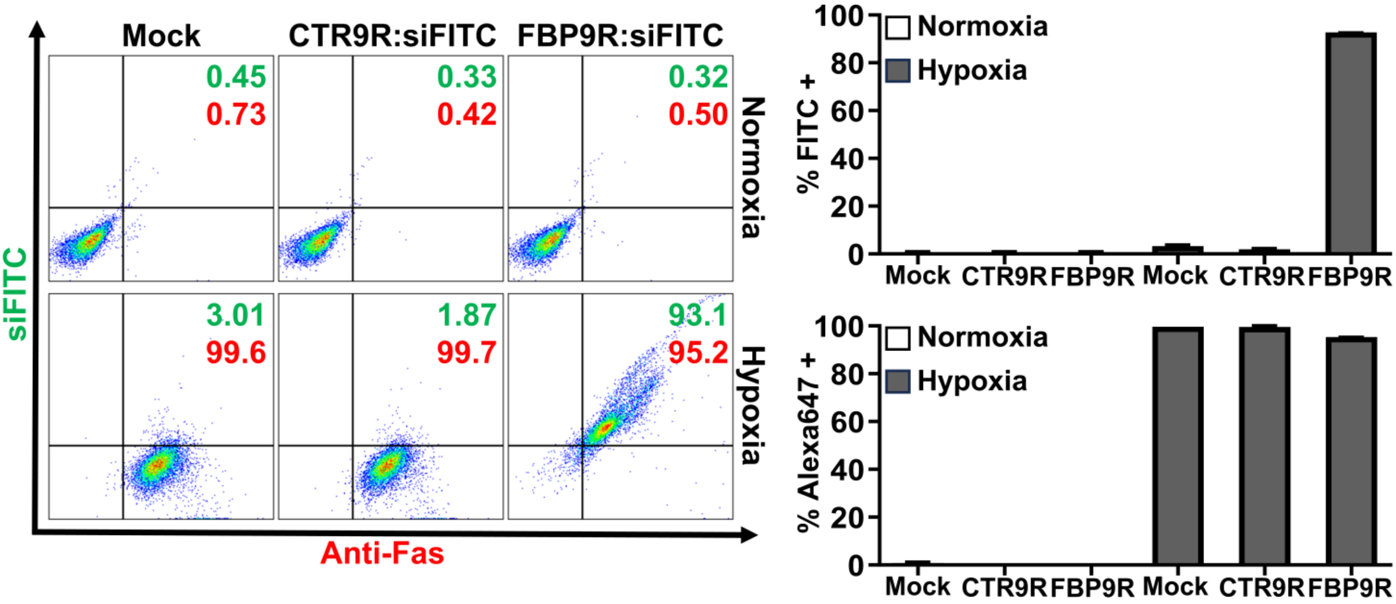


**Figure S3. Fas induction in Neuro-2a cells under hypoxic conditions.** Flow cytometry analysis for Fas and FBP9R binding in hypoxia Neuro-2a cells. Hypoxia induced Fas and FBP9R binding exclusively to hypoxic cells. In hypoxia, scrambled peptide (CTR9R) did not bind to Fas, although cells expressed Fas. Representative dot-plot (left panel) and cumulative data for percent of Fas-positive cells (right panel). *p<0.05, **p<0.01 versus mock group. All data were obtained from three independent experiments and shown as mean ± SD.

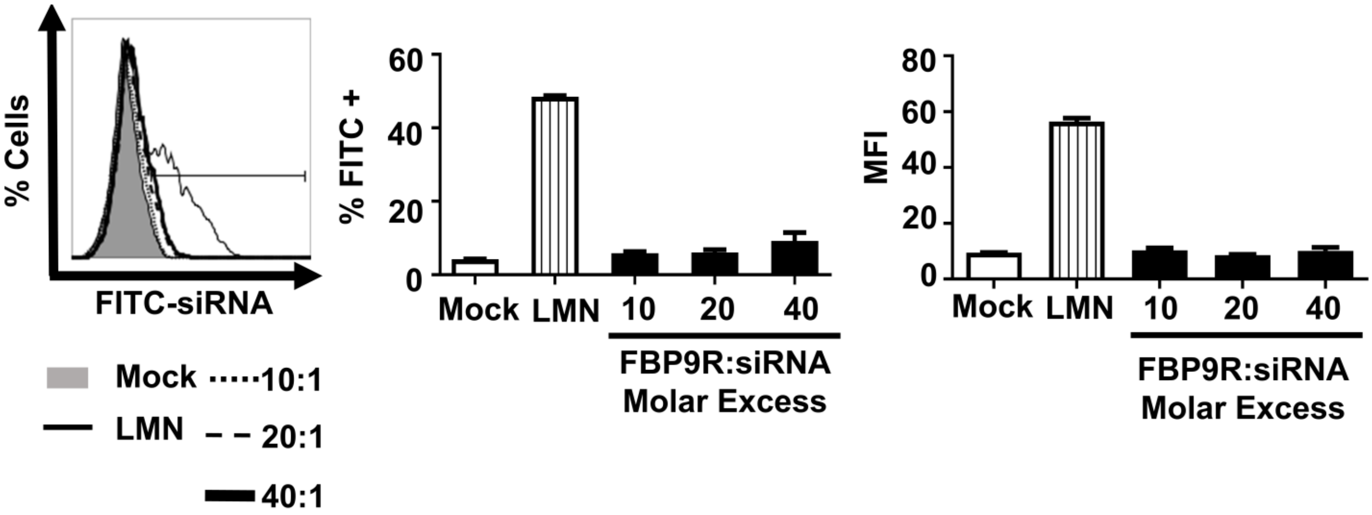


**Figure S4. Ineffectiveness of FBP9R in delivering siRNA into normoxia Neuro-2a cells.** Flow cytometry analysis for the efficacy of siRNA delivery by FBP9R/siRNA nanocomplexes in hypoxia Neuro-2a cells. FBP9R peptides were complexed with 200 pmole of FITC-labeled siRNA at the indicated peptide:siRNA molar excess. Representative histogram (left panel) and cumulative data for percent of FITC-positive cells (middle panel), mean fluorescence intensity (right panel). *p<0.05, **p<0.01 versus mock group. All data were obtained from three independent experiments and shown as mean ± SD.

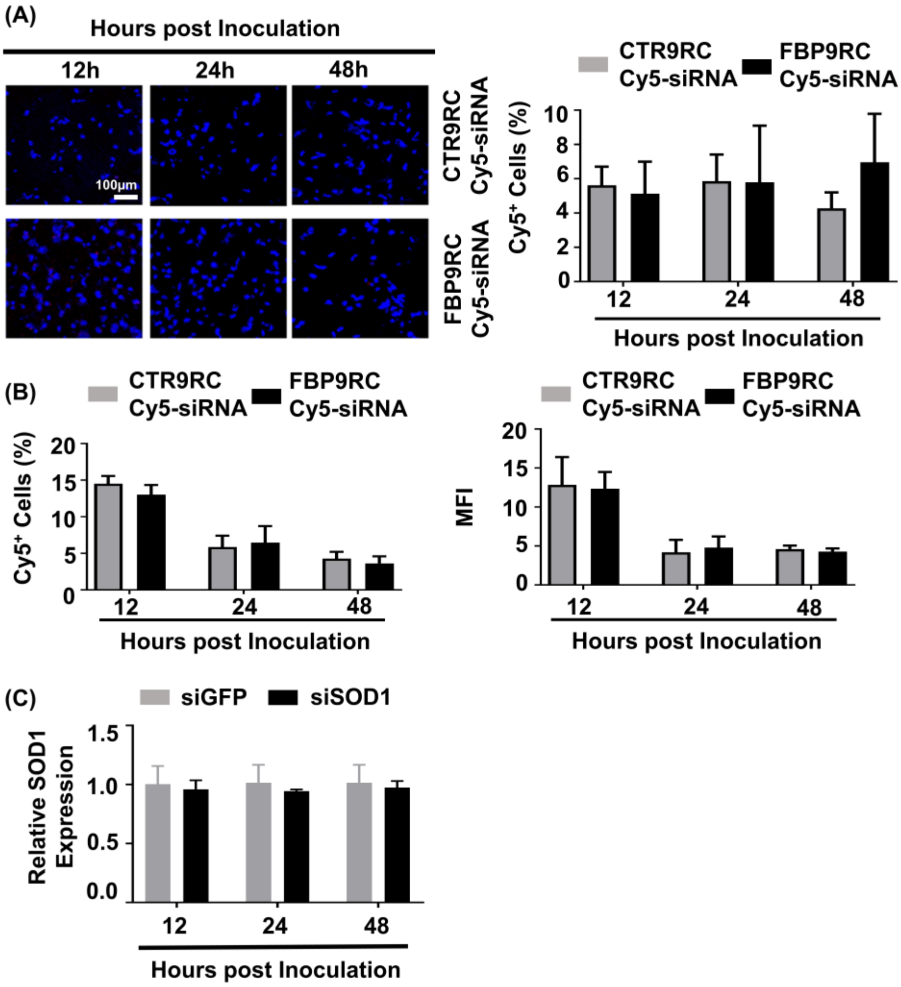


**Figure S5. Lack of localization of intranasally delivered FBP9R/siRNA nanocomplexes to normoxia brain regions in the rat model of brain ischemia.** (A) Immunohistochemistry of intranasally delivered Cy5-labeled siRNA in the normal hemisphere at indicated hours post-injection. Representative images (left panel) and cumulative data from six independent animals per groups (right panel) indicating percent of Cy5-positive cells. Scale bar indicates 50 μm. (B) Flow cytometry analysis of Cy5-positive cells in the infarcted hemisphere. Cumulative data for percent of Cy5-positive cells (left panel) or mean fluorescence intensity (right panel) obtained from six independent animals per groups. (C) Gene silencing efficiency of intranasally delivered FBP9R/siRNA nanocomplexes in the normal hemisphere from six independent animals.
